# Reply to the letter from Dr. Miao et al.

**DOI:** 10.14814/phy2.12964

**Published:** 2016-09-15

**Authors:** Yuko Tanimura, Wataru Aoi, Yoshikazu Takanami, Yukari Kawai, Katsura Mizushima, Yuji Naito, Toshikazu Yoshikawa

**Affiliations:** ^1^ Department of Molecular Gastroenterology and Hepatology Kyoto Prefectural University of Medicine Kyoto Japan; ^2^ Faculty of Human Aichi Toho University Nagoya Japan; ^3^ Laboratory of Health Science Graduate School of Life and Environmental Sciences Kyoto Prefectural University Kyoto Japan; ^4^ Department of Home Economics Otsuma Women's University Tokyo Japan; ^5^ Louis Pasteur Center for Medical Research Kyoto Japan

We appreciate the comments and insights provided by Dr. Miao in response to our article (Tanimura et al. 2016), and thank the Editor for allowing us to reply.

We showed that acute exercise increased the circulating FGF21 concentrations of human and mice. In the exercise condition, FGF21 secreted from liver would affect the circulating level of FGF21 more than muscle‐secreted FGF21. However, we discussed that muscle‐derived FGF21 may rather mainly act though paracrine and autocrine effects in muscle tissues locally, which contributes an improvement of whole body metabolism via activating metabolism of muscle.

First, Miao et al. indicated that FGF21 resistance might be improved by the acute exercise, which would be a convincing point. As a similar example, circulating adiponectin was increased by exercise in obese state (Kondo et al. 2006); although the expression of adiponectin receptor was also increased by acute exercise (Huang et al. 2007), which leads to an improvement of adiponectin resistance and is a mechanism of exercise‐induced metabolic improvement. In contrast, the previous study (Fisher et al. 2010) showed that the expression of FGF21 receptor was decreased with obese development, which results in an increase in the circulating levels of FGF21. We agree that exercise‐induced metabolic improvement can be partly mediated by increasing the expression in FGF21 receptor, as Dr. Miao suggested. However, subjects in our study were not obese; thereby, we assume that the increase of circulating FGF21 simply contributed to metabolic improvement. Additionally, the improvement of FGF21 resistance might be not mediated only through the receptor, just like the improvement of insulin resistance by exercise.

Next, they pointed out that excessively upregulated FGF21 may induce a vicious loop of FGF21 resistance. In this regard, we consider that the condition of transient elevation should be separated with the condition of chronic elevation. Indeed, the chronic increase of FGF21 in obesity induced FGF21 resistance (Giralt et al. 2015). Additionally, circulating FGF21 in obesity was not changed by exercise training, regardless of the improvement of metabolism (Besse‐Patin et al. 2014). Furthermore, the degree of circulating FGF21 elevation by acute exercise was smaller in obesity than lean (Slusher et al. 2015), suggesting that it is difficult to obtain additive increase in the condition of high FGF21 level. Therefore, we discussed on the basis of the concept that the transient elevation induced by exercise has different function and regulation from chronic elevation in pathological states. However, further studies will be needed to investigate the expression of FGF21 receptor by acute exercise.
